# Novel Approaches for Investigating Host Responses to HIV Infection in Ex Vivo Human Genital Tissues

**DOI:** 10.1111/aji.70240

**Published:** 2026-04-29

**Authors:** Mathias Franzén Boger, Vilde Kaldhusdal, Kristina Broliden

**Affiliations:** ^1^ Department of Medicine Solna Division of Infectious Diseases Karolinska Institutet Department of Infectious Diseases Center For Molecular Medicine Karolinska University Hospital Stockholm Sweden

**Keywords:** ex vivo tissue models, genital mucosa, HIV, host immune response, mucosal barrier, multiplex imaging, spatial transcriptomics

## Abstract

Understanding local immune responses and the structural integrity of the genital mucosal barrier is essential for advancing knowledge of both susceptibility to sexual HIV transmission and the pathogenesis of chronic HIV infection. This review highlights innovative methodologies for investigating host responses to HIV infection using ex vivo human genital tissues. Recent advances in spatial transcriptomics and multiplex imaging enable high‐resolution analysis of tissue architecture, immune cell distribution, and gene expression within mucosal environments. These technologies reveal spatially heterogeneous immune responses as well as epithelial and submucosal structural alterations associated with increased HIV susceptibility and chronic infection. Computational workflows tailored to genital tissue morphology enhance reproducibility and support the integration of transcriptomic and imaging data. Despite current limitations, such as resolution constraints and high costs, ongoing improvements in spatial transcriptomics and bioimaging platforms promise deeper insights into mucosal barrier function and HIV pathogenesis. Characterizing tissue‐specific immune dynamics through these approaches may guide the development of targeted interventions aimed at reinforcing mucosal defenses and reducing vulnerability to HIV infection.

## 1 Introduction

In the early decades of HIV research, scientific focus predominantly centered on blood‐derived samples. This approach overlooked mucosal tissues, particularly those in the female reproductive tract, which are the primary sites of viral entry during sexual transmission. A parallel can be drawn from the coronavirus disease 2019 (COVID‐19) pandemic, which underscored that immune responses in peripheral blood may differ substantially from those at mucosal tissue sites where pathogens initiate replication and drive disease processes [1, 2]. HIV utilizes specialized immunological features of mucosal environments to establish infection, including their cellular composition, cytokine milieu, and epithelial barriers [3, 4]. Lymphoid tissues associated with mucosal sites, especially the gut, are particularly susceptible to HIV infection due to their abundance of activated CD4+ T lymphocytes [5, 6]. Approaches that dissect how systemic, mucosal, and lymphoid compartments differ in their immunological dynamics are central to advancing prevention, therapeutic, and vaccine strategies against HIV.

Investigations of gut‐associated lymphoid tissues have illuminated several mechanisms underlying HIV pathogenesis, such as epithelial integrity breakdown, profound CD4+ T cell depletion, and chronic immune activation [7]. In contrast, the female reproductive tract has been comparatively underexplored during the acute and chronic phases of HIV infection. The lack of information reflects persistent barriers: limited access to clinical specimens, ethical and procedural constraints, and the complexity of immune networks within the reproductive tract. Consequently, our understanding of local host defenses, establishment of viral reservoirs, and tissue‐specific progression at this site remains incomplete, hindering interventions designed to block infection.

Future studies should prioritize the acquisition of cervicovaginal tissue specimens from women across distinct stages of HIV infection, including acute and chronic disease, as well as from seronegative women with elevated exposure risk. Access to such material would enable detailed characterization of the immune milieu and tissue architecture at the main site of viral entry; it would also provide long‐term persistence insights. Although non‐human primate models have advanced the field, there remain fundamental distinctions between species, including variations in mucosal immune organization, endocrine influences, microbial communities, and tissue structure. These differences restrict the direct applicability of animal‐derived observations to human biology. In contrast, ex vivo experimentation with human mucosal tissue offers a uniquely relevant framework to investigate processes that are specific to human infection, host defense, and therapeutic response.

Recent technological advances have transformed the field of human mucosal immunity and spatial biology of HIV infection [8, 9]. High‐dimensional approaches such as spatial transcriptomics, spatially resolved proteomics (e.g., multiplex immunofluorescence imaging), single‐cell RNA sequencing, and live‐cell imaging combined with functional assays now permit detailed analysis of tissue microenvironments. When applied to the upper female genital tract, spatial transcriptomic profiling of the human fallopian tube epithelium has revealed spatially regulated gene expression patterns with important implications for female reproductive health [10]. These spatial omics approaches are also particularly valuable for studying the lower female genital tract mucosa, which is composed of the vulva, vagina and ectocervix. Each of these compartments are defined by distinct epithelial structures, resident microbiota, and immune cell populations. The methods can generate integrative datasets that capture cellular diversity and spatial context with precision unattainable by conventional histology or bulk analyses. In this review, we describe the application of multiplex imaging and spatial transcriptomics to ex vivo ectocervical tissues obtained from women with established HIV infection, as well as individuals with elevated risk. Through these examples, we emphasize both the novel insights yielded and the broader promise of such approaches for elucidating host‐pathogen interactions and maintenance of mucosal barrier function in HIV disease.

## 2 Ethical and Logistical Considerations in Tissue Procurement and Data Handling

### 2.1 Ethical and Practical Dimensions of Obtaining Female Reproductive Tract Biopsies

The collection of ectocervical and vaginal tissue specimens in populations heavily affected by HIV requires careful ethical deliberation. Procedures of this nature are inherently invasive and must respect participant safety, autonomy, and cultural sensitivities. Chief concerns include ensuring informed consent, protecting participants during the recovery period, and minimizing behavioral or clinical risks. International guidance documents (e.g., those issued by UNAIDS and the World Health Organization) emphasize key principles for ethically conducting such research [11, 12].

A notable example was provided by Lajoie et al. [13], who conducted biopsies within a Kenyan cohort of female sex workers. The study illustrates how ethical challenges can be addressed through integrated biomedical, behavioral, and community‐based safeguards. A comprehension tool was developed to confirm that participants fully understood both the biopsy procedure and its implications, including the need to abstain from sexual activity during healing. Recognizing that mucosal injury could temporarily increase vulnerability to HIV acquisition, adherence can be monitored by testing cervicovaginal secretion samples for prostate‐specific antigen, a commonly used biomarker of vaginal semen exposure [14]. Discreet mobile phone reminders can further support post‐procedure compliance and financial reimbursement can be framed not as coercion but as fair compensation for income lost due to required abstinence. Collaborative efforts with local clinicians and peer educators can further strengthen trust and ensure cultural sensitivity.

From a methodological perspective, two ectocervical punch biopsies of approximately 2–3 mm, paired with cervicovaginal secretion samples, are typically sufficient to support multi‐omics analyses of gene and protein expression. For transcriptomic studies, one biopsy specimen should immediately be placed in RNA‐preserving medium. The second biopsy may be snap‐frozen or fixed in formaldehyde, depending on plans for imaging or proteomic applications. These collection practices adhere to standard clinical procedures for cervical dysplasia screening. Vaginal biopsies usually require local anesthesia but otherwise follow a similar approach.

### 2.2 Ethical Considerations in Spatially Resolved High‐Dimensional Data

The advent of spatial omics methods has enabled close examination of tissue architecture and molecular dynamics [15]. However, application of these approaches to ex vivo genital tract samples could introduce ethical challenges because spatial information can inadvertently capture unique anatomical or pathological features. Unlike bulk omics, the resolution of spatial datasets may hinder effective anonymization; rare conditions or small cohorts further increase the risk of re‐identification when combined with contextual metadata. Informed consent procedures must therefore explicitly explain the scope of spatial data generation.

The broader movement toward open science must be carefully balanced with responsible governance of sensitive spatial datasets. Open‐access repositories may be unsuitable for unrestricted deposition of genital tissue data. Instead, controlled‐access infrastructures can offer models for secure distribution with strict usage conditions [16, 17]. Additional data‐sharing mechanisms emphasize the importance of harmonized ethical frameworks and governance structures for secondary data use.

## 3 Advanced Bioimaging and Spatial Omics for the Study of Mucosal Barriers and Immunity

### 3.1 Multiplex Immunofluorescence for High‐Dimensional Tissue and Immune Profiling

**FIGURE 1:**
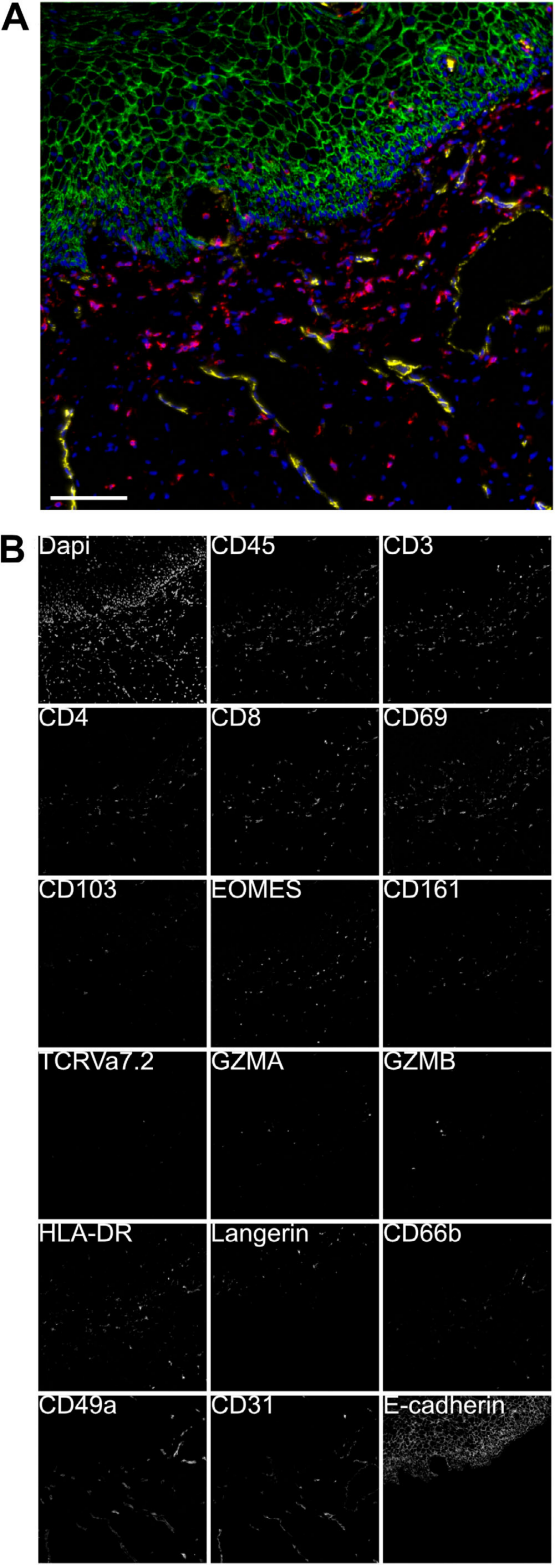
Leukocyte identification using multiepitope ligand cartography (MELC) in a human ectocervical epithelium from an ex vivo genital tissue sample obtained from a Kenyan female sex worker within our clinical study cohort. A multiplex staining panel was applied. (A) Nuclear staining (DAPI, blue), leukocytes (CD45, red), epithelial structures (E‐cadherin, green), and blood vessels (CD31, yellow) are visualized. (B) In the same region as depicted in (A), the full MELC marker panel (DAPI, CD45, CD3, CD4, CD8, CD69, CD103, EOMES, CD161, TCRVa7.2, GZMA, GZMB, HLA‐DR, Langerin, CD66b, CD49a, CD31, E‐cadherin) used for tissue profiling is displayed.

Digital bioimage analysis involves the systematic extraction of reproducible, quantitative information from digitalized tissue or cellular images (Figure 1). Over the past two decades, the field has shifted from subjective, manual interpretation toward highly automated, standardized approaches. A major turning point occurred with the introduction of whole‐slide imaging in the early 2000s, which made histological material broadly accessible to researchers beyond specialized pathology laboratories [18].

In biomedical research, digital imaging methods are now integral to the study of cellular organization, tissue structure, and therapeutic distribution [19, 20, 21]. Clinical analyses have also experienced substantial progress, particularly in oncology. Over the past decade, the United States Food and Drug Administration has approved bioimaging solutions for diagnostic applications, including automated scoring systems for breast cancer [22]. Furthermore, Ström et al. applied an artificial intelligence‐based image analysis pipeline to prostate cancer diagnostics and grading [23]. This is applicable to HIV research, where quantitative histological assessment of lymphoid and mucosal tissue is essential for understanding viral reservoirs, immune activation, and tissue‐specific pathology.

Advanced multiplex immunofluorescence is a robust aspect within the emerging field of spatial proteomics, recognized as Nature Methods’ “Method of the Year 2024” [24]. This field permits simultaneous detection of dozens to hundreds of protein markers within intact mucosal tissue sections, thereby capturing spatial relationships within the local immune landscape. Commercial systems supporting multiplex imaging are now widely available, offering unprecedented opportunities to interrogate the complex cellular and molecular dynamics underlying infection, inflammation, and tissue homeostasis.

### 3.2 Spatial Transcriptomics to Characterize Mucosal Architecture and Immune Dynamics

Single‐cell RNA sequencing (scRNA‐seq) has transformed biomedical research by providing detailed transcriptional profiles at the individual cell level. However, this approach has inherent limitations: the required tissue dissociation can induce cellular stress, alter gene expression patterns, and eliminate the spatial context needed to understand tissue organization and intercellular communication.

**FIGURE 2:**
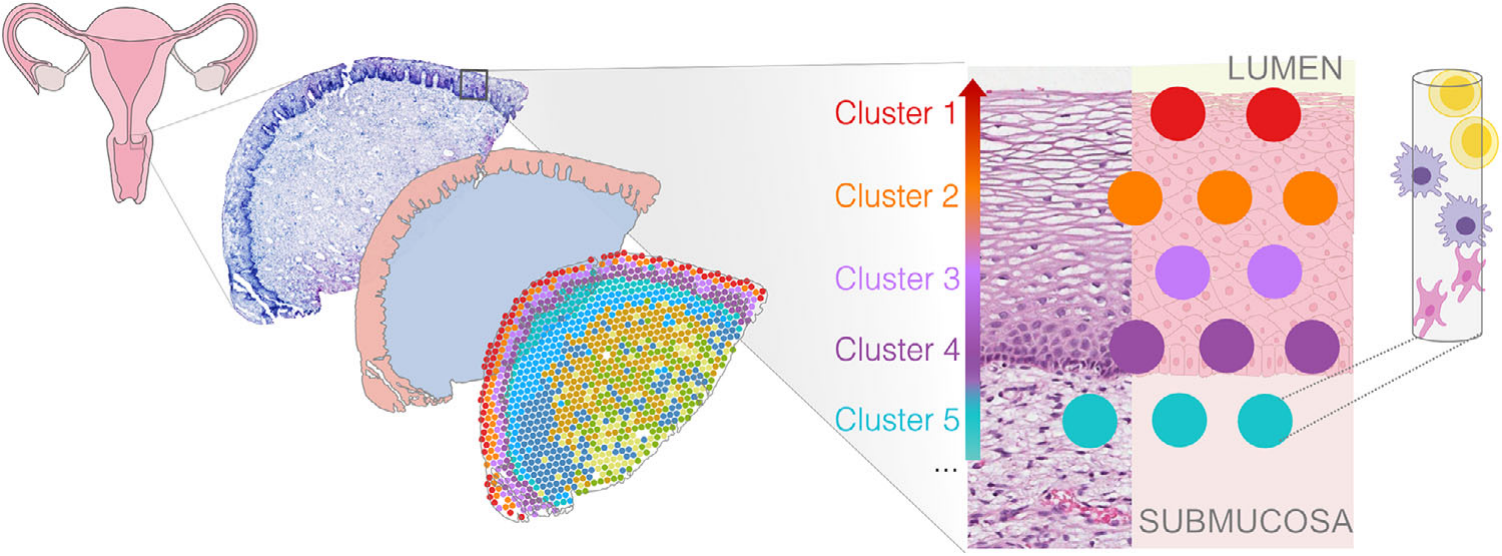
Schematic illustration of spatial transcriptomics applied to ectocervical tissue. Anatomical localization of the ectocervix (left), with a histological section used for spatial transcriptomics analysis. Transcriptome capture locations are represented by spots, which are grouped into transcriptionally defined clusters. These clusters correspond to distinct epithelial layers and submucosal regions, as shown in the histological reference (middle). The schematic (right) illustrates that each spot may contain multiple cells and cell types.

A breakthrough occurred in 2016, with the introduction of a methodology for spatially resolved transcriptomics [15], later commercialized as Visium by 10x Genomics. This innovation enabled gene expression to be quantified directly in intact tissue sections, linking molecular information with histological structure. By preserving positional context, spatial transcriptomics allowed researchers to examine how gene expression varies across tissue regions and how cells interact in their native microenvironments (Figure 2).

Although spatial transcriptomics typically achieves lower resolution relative to scRNA‐seq, the two approaches are complementary. scRNA‐seq provides robust transcriptional heterogeneity insights, whereas spatial transcriptomics anchor these data to specific tissue locations. Integration is typically achieved through one or more computational strategies, including using scRNA‐seq as a reference to infer cell types in each spatial spot as well as using various spatial interaction analyses to characterize microenvironments and communication networks [25].

Recent advances in spatial transcriptomics have facilitated higher sensitivity, greater multiplexing, and finer resolution [26]. Emerging platforms support the construction of spatially resolved tissue atlases encompassing thousands of simultaneously detected transcripts. These capabilities are particularly useful in studies of infectious diseases such as HIV, where the design of targeted therapeutic strategies relies on knowledge of how viral reservoirs, immune populations, and tissue remodeling processes are distributed across genital mucosa.

### 3.3 Toward a Multiomics Atlas of the Female Reproductive Tract

Although Interest in Mucosal Immunology and Infectious Disease Research Has Expanded Considerably, Publicly Available Datasets for the Female Genital Tract Remain Heavily Weighted Toward Cancer. Large‐scale Efforts Such as the Cancer Genome Atlas (TCGA) and the Cancer Research Data Commons (CRDC) Have Generated Extensive Datasets for Cervical, Vaginal, and Vulvar Carcinomas [27, 28]. In Contrast, Datasets Derived From Non‐malignant Tissues, Particularly From Healthy Women or Those Affected by HIV, Are Scarce. This Imbalance Restricts Comparative Investigations Across Physiological and Pathological States, Anatomical Regions, and Demographic Groups. It Also Limits Progress in Modeling Mucosal Barrier Function, Host Defense, and Susceptibility to Sexually Transmitted Infections

In Addition to the Human Protein Atlas [29], a Spatially Resolved Multiomics Atlas of the Female Genital Tract Could Provide Transcriptomic, Proteomic, and Imaging Datasets Essential for Advancing the Field. Recent Studies Underscore the Feasibility and Utility of this Approach. For Example, Analyses of Cervical Tissue From Women Living With HIV Have Revealed Epithelial Barrier Disruption, Chronic Immune Activation, and Altered Positioning of CD4+ Target Cells, Processes central to both Transmission and Pathogenesis [30, 31]. A Comprehensive Atlas Could Incorporate Multiple Design Features: Inclusion of both Healthy Volunteers and Participants Living With HIV; Longitudinal Sampling to Capture Dynamic Changes Over Time; and Standardized Metadata Documenting Clinical Characteristics, Hormonal Influences, and Microbial Composition

## 4 Data Analysis and Computational Workflows

### 4.1 Software Platforms and Core Concepts in Bioimage Analysis

Progress in imaging hardware and computational techniques has been accompanied by the development of numerous digital image analysis softwares. Commercial packages, reliable and often bundled with specific imaging systems, tend to prioritize standardization over flexibility. Their proprietary nature can limit adaptation to specialized studies or exploratory investigations. To address these limitations, biomarkers of HIV susceptibility can be characterized within genital tissues via open‐source software platforms, such as QuPath [32], FIJI/ImageJ [33], CellProfiler [34], and Ilastik [35]. These platforms provide a versatile environment for tailoring workflows to the requirements of projects. Once a workflow is established and validated, the platforms may enable: a) Automated processing of large image datasets with consistent parameters, b) Significant time savings and reduced manual intervention for high‐throughput studies, c) Enhanced reproducibility through standardized automated workflows, and d) Scalability for studies involving hundreds or thousands of images. In our research, some of them supported the design of customized workflows for compartment‐specific (e.g., multistratified epithelium or submucosa) image segmentation, immune cell enumeration, and evaluation of epithelial barrier integrity—analyses that would have been challenging to implement with fixed commercial frameworks. Moreover, the workflows can be partially reused between tissues with similar histological structures, allowing for efficient adaptation and reproducibility of analysis across related sample types.

An additional benefit of open‐source software is the ease of disseminating protocols to the broader research community, which enhances transparency, reproducibility, and collective refinement. These workflows are not restricted by licensing fees or proprietary constraints. Their adaptability also makes them suitable for multiple tissue types and experimental settings. However, the advantages of open‐source software also carry certain challenges. Effective use typically demands greater technical expertise, which can hinder researchers unfamiliar with image analysis. Furthermore, the development of validated, project‐specific workflows can be resource‐intensive. Pipelines must be optimized, and accuracy must be confirmed before results can be interpreted with confidence. Another important consideration is a high computational demand, especially when processing large image files and data sets. This necessitates either capable workstations with sufficient processing power or use of cloud‐based analysis platforms capable of providing adequate graphics processing unit (GPU) resources. Regardless of the software environment, bioimage analysis workflows can comprise the following core steps: (a) image pre‐processing to mitigate background noise and correct color intensity variations, (b) segmentation to define objects of interest, such as tissue compartments or individual cells, (c) extraction of descriptive features such as area and fluorescence intensity, and (d) classification of results, including shape, size, or signal intensity [36].

### 4.2 Computational Environments for Spatial Transcriptomics

Spatial transcriptomic data interpretation requires the integration of molecular readouts with the underlying histological framework. The value of these datasets is realized only when visualizations are clear and scalable across single or multiple tissue sections. To support such analyses, diverse computational tools have been developed in common programming environments such as R and Python. Among the most widely applied frameworks, Seurat (implemented in R; [37] was originally designed for single‐cell transcriptomic studies but has since been extended to accommodate spatial technologies. Scanpy [38] provides comparable functionality in Python, offering pipelines tailored for various single‐cell and spatial data analyses. A spatial transcriptomics data analysis workflow can include the following core steps: (a) importing data, (b) quality control, (c) sample integration and dimensionality reduction, (d) clustering and cluster annotation (differential gene expression analysis of clusters), (e) differential gene expression across study groups, (f) deconvolution of spots into percent cell types [39].

### 4.3 Replicates in Spatial and Imaging‐Based Analyses

The utilities of spatial omics and imaging platforms heavily depend on the reliability of their outputs, which are shaped by the quality of biological and technical replicates. Biological replicates, drawn from distinct donors or anatomical sites, exhibit substantial variability due to differences in age, hormonal milieu, microbiome composition, and disease status. This biological diversity can interact with technical sources of variation introduced during sample collection and processing. Mucosal samples from the female reproductive tract can be sensitive to both enzymatic degradation and mechanical stress. Procedural differences (e.g., biopsy technique, transport conditions, and fixation methods) can substantially alter tissue morphology and molecular integrity. Downstream spatial proteomics and transcriptomics readouts can be influenced by pre‐analytical factors, including freezing delay, embedding medium (optimal cutting temperature compound (OCT) versus paraffin), and section thickness, each of which can affect staining performance and RNA preservation.

Spatial profiling platforms, such as 10x Genomics Visium/Xenium, NanoString CosMx, and multiplex immunofluorescence, present distinct strengths and limitations that influence interpretation and comparison of their results. Immunofluorescence‐based approaches heavily depend on antibody performance, fluorophore stability, and degree of autofluorescence, which can differ across tissues and experimental runs. Batch effects may be introduced by variations in reagent lots, scanner calibration, or imaging parameters [40]. Transcriptomic platforms differ in spatial resolution and transcript capture efficiency. For example, Visium provides broad transcriptome coverage with moderate spatial detail, whereas CosMx achieves single‐cell resolution using targeted panels [41, 42]. These distinctions must be considered when analyzing results across platforms or attempting data integration.

Reproducibility in spatial studies requires careful design. The inclusion of multiple biological replicates—such as samples from different individuals or anatomical regions—together with technical replicates, such as repeat staining or sequencing, allows sources of variability to be identified and quantified. The feasibility of incorporating multiple replicates is however often limited by the invasive nature of tissue collection and the resulting scarcity of available samples. In addition, the high cost associated with these methodologies poses significant barriers to repeating experiments. Key quality metrics such as tissue coverage, RNA integrity number, signal‐to‐noise ratios, and segmentation accuracy should be routinely reported and used to filter low‐quality data. For cross‐platform analyses, normalization and batch correction strategies (e.g., Harmony, Seurat‐based approaches) are essential to minimize technical artifacts while retaining true biological signals.

## 5 Application of Multiplex Imaging and Spatial Transcriptomics in Human Genital Tissues

### 5.1 Tailored Bioimage Analysis Workflows for High‐resolution Characterization of Mucosal Tissues

Studies in non‐human primates have demonstrated that epithelial integrity is a major determinant of susceptibility to SIV [43, 44]. A strong epithelial barrier in the female reproductive tract may theoretically contribute to the often relatively inefficient transmission of HIV. However, epithelial characteristics substantially differ across anatomical sites. In the vagina and ectocervix, the surface epithelium is typically 150–500 µm thick and comprising 25–30 layers of stratified squamous cells. In contrast, the rectal mucosa consists of a single columnar epithelial layer. This monolayer is likely to offer less mechanical defense and is vulnerable to microtears during anal intercourse, potentially facilitating viral entry.

To obtain reliable and reproducible information from mucosal tissues of varying complexity, image analysis workflows specifically adapted to the structural features of each site are needed. In a phase I human clinical trial evaluating the antiviral compound Griffithsin, we developed an automated pipeline for rectal biopsies characterized by a single‐layer epithelium. The method separated epithelial and submucosal compartments, quantified CD4^+^ lymphocytes, and assessed epithelial integrity via E‐cadherin staining [45]. Segmentation routines were optimized to reflect the morphology of columnar epithelia, ensuring unbiased analysis across multiple specimens.

We applied a similar approach to the multi‐layered stratified squamous epithelium of the ectocervix [46]. Our workflow was modified to improve segmentation and enhance quantification of structural disturbances linked to chronic HIV infection [30]. A validated approach for enumerating CD4^+^ cells in ectocervical tissues was utilized during both projects, ensuring methodological consistency and facilitating comparisons [47]. Extensions of these protocols have been utilized in studies of male genital tissues to identify biomarkers associated with HIV acquisition risk [48].

Furthermore, we revised bioimage analysis workflows to handle high‐dimensional datasets generated by multiplex immunofluorescence, including Multi‐Epitope Ligand Cartography (MELC) [31]. Incorporating 18 immune markers, this data‐driven approach enabled detailed phenotypic and spatial characterization of cervical immune responses in the context of chronic HIV infection. Advanced clustering algorithms and dimensionality reduction techniques were utilized to delineate immune cell populations and define their spatial arrangements within the tissue microenvironment.

Taken together, these tailored computational workflows highlight how automated segmentation, marker quantification, and spatial mapping can be integrated to uncover tissue‐specific immune dynamics and structural alterations associated with HIV pathogenesis. Such approaches provide a robust framework for high‐resolution analysis of mucosal tissue.

### 5.2 Spatial Transcriptomics Identifies Compartment‐level Alterations in Gene Expression Affecting Genital Barrier Integrity

Conventional tissue‐disruptive methods such as flow cytometry and scRNA‐seq have yielded valuable insights concerning the effects of HIV on immune mediators and epithelial function. However, these approaches have limitations. For instance, Steinert et al. reported discrepancies between immune cell frequencies measured in dissociated mucosal suspensions and those observed by microscopy, suggesting that essential spatial information is lost during tissue processing [49]. These findings highlight the importance of approaches that maintain structural context when investigating mucosal immunity in HIV infection.

Our recent work has shown that chronic HIV infection is linked to localized transcriptional changes within the genital mucosa [30, 31]. Genes involved in epithelial junctional complexes, extracellular matrix stability, and barrier integrity were downregulated in discrete regions of the ectocervix. Such alterations may create microenvironments more permissive to pathogen entry and immune cell infiltration. These results emphasize that epithelial disruption in HIV infection is not uniform but spatially heterogeneous, underscoring the need for tissue‐resolved analyses.

Spatial biology techniques have also proven useful for studies on how the local microbiome influences the immune environment in the female reproductive tract [50, 51, 52]. When comparing women with a highly diverse cervicovaginal microbiome to those with a *Lactobacillus crispatus/acidophilus*‐dominated microbiome, spatial transcriptomics revealed a distinct gene expression profile localized to the epithelial basal membrane of the ectocervix [53]. The differentially expressed genes in the former group were associated with epithelial maintenance, submucosal extracellular matrix organization, and immune function. In contrast, women with a *Lactobacillus crispatus*/*acidophilus*‐dominated microbiome exhibited a gene expression profile enriched for pathways that support mucosal barrier integrity throughout the epithelial layers.

In another study, Kaldhusdal et al. [39] demonstrated that hormonal modulation further shapes the ectocervical immune environment. Ex vivo tissues from women using depot medroxyprogesterone acetate (DMPA) exhibited broad upregulation of immunoglobulin transcripts along with localized suppression of genes critical for epithelial and matrix integrity. These patterns were linked to systemic estradiol levels, suggesting that DMPA‐induced hypoestrogenemia impacts mucosal integrity and modulates immune responses in a compartment‐specific manner. Through unsupervised clustering to distinguish epithelial and submucosal compartments, the study provided a high‐resolution map of tissue architecture and immune activity. Similarly, Parthasarathy et al. [54] characterized genital cell populations at single‐cell resolution and examined how dendritic cell subsets respond immediately following HIV exposure. The earliest antiviral response was dominated by dendritic cells, marked by rapid cytokine secretion, activation of non‐classical inflammatory pathways, and induction of host restriction factors. Collectively, these findings illustrate how spatial transcriptomics and single‐cell approaches can uncover compartment‐specific shifts in gene expression that influence genital barrier function and HIV susceptibility.

### 5.3 Combined Insights from Multiplex Imaging and Spatial Transcriptomics in HIV Research

**FIGURE 3:**
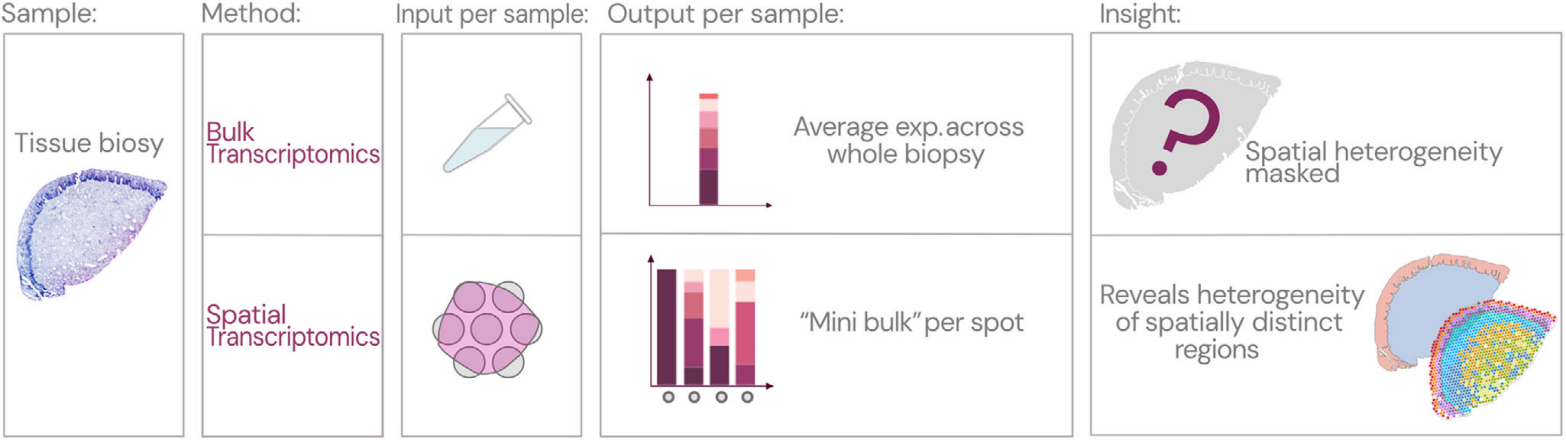
Bulk versus spatial transcriptomics in tissue biopsies. Comparison of bulk and spatial transcriptomics, highlighting differences in sample input, data output, and spatial resolution. Bulk transcriptomics averages gene expression across the tissue, while spatial transcriptomics preserves spatial context and reveals regional heterogeneity.

Bulk RNA sequencing has advanced understanding of mucosal immunity, but its reliance on population averages masks the spatial context of cell‐cell interactions (Figure 3). Spatial transcriptomics addresses this limitation by preserving tissue architecture; in conjunction with multiplex imaging, it provides an integrated view of transcriptional and translational features within intact mucosa. This combined approach enables concurrent mapping of structural barriers and immune cell populations, creating a multidimensional portrait of the HIV‐associated mucosal microenvironment.

There is increasing evidence that resident immune cells within non‐lymphoid tissues exhibit functional properties distinct from their circulating counterparts [55]. This observation underscores the importance of studying tissue‐resident immune cells to clarify mechanisms of HIV pathogenesis. Insights into the positioning of target and effector leukocytes within genital tissues are critical for efforts to elucidate the local immune response to HIV infection [56, 57]. Nevertheless, many studies have relied on indirect sampling strategies such as cervicovaginal lavage or cytobrush collection, which lack spatial resolution [58, 59]. Recently, our spatially resolved analyses of ectocervical tissue from Kenyan female sex workers have demonstrated broad immune activation extending across both epithelial and submucosal layers [31, 39]. Elevated expression of genes associated with interferon signaling and humoral responses reflected a chronic antiviral state; transcripts linked to T cell effector functions appeared in more spatially restricted regions compared with seronegative controls. These observations suggest that HIV infection induces compartmentalization rather than uniform immune modulation. This spatial heterogeneity may explain how chronic infection impacts mucosal immunity and could influence viral persistence or immune exhaustion [60, 61].

The combined insights of imaging‐based and transcriptomic datasets provides a comprehensive strategy to characterize mucosal immunity in women living with HIV and women with an elevated risk of HIV acquisition [31]. These complementary methodologies have thus revealed pronounced epithelial disruption and altered immune regulation in ex vivo‐derived ectocervical tissue samples from women who are HIV‐seropositive. The findings provide critical insights into the mechanisms of HIV pathogenesis and the associated enhanced susceptibility to other sexually transmitted infections.

## 6 Current Limitations and Future Prospects

### 6.1 Limitations and Potential Improvements in Genital Mucosal Tissue Sampling

Major challenges in invasive genital mucosal tissue sampling include participant recruitment, limited access to medical facilities and trained personnel, requirements for participants to abstain from unprotected sexual activity for a period before and after the procedure, and the lack of standardized protocols for sampling and analysis [62, 63]. Variability in sample collection methods, tissue processing, fixation, and storage can introduce significant technical variability. Moreover, differences in the selection of anatomical sites, staining panels, and analytical pipelines hinder cross‐study comparisons and limit the reproducibility of findings. Global efforts to harmonize mucosal sampling protocols can enhance reproducibility and facilitate cross‐study comparisons. Furthermore, the development of open‐access repositories for validated protocols, reagents, and metadata standards would support broader adoption and transparency.

The high cost of reagents, instrumentation, and computational infrastructure also remains a significant limitation [64]. In addition, the large and complex datasets generated by these spatially resolved techniques demand advanced bioinformatics capabilities. As the technologies mature, reductions in cost and improvements in throughput are expected. The development of user‐friendly, open‐source bioinformatics pipelines and machine learning algorithms can further streamline data analysis and interpretation.

### 6.2 Limitations and Potential Improvements of Multiplex Immunofluorescence

Analyses based on multiplexed immunofluorescence typically yield two‐dimensional projections, although the tissues themselves are three‐dimensional. This limitation can obscure important features such as the continuity of junctional proteins or the precise localization of immune cells along the z‐axis. For example, disruption of epithelial junctions observed in a single optical plane may not accurately represent network integrity throughout the tissue. Similarly, immune cells appearing close to the epithelial boundary in two‐dimensional images may reside within a different spatial layer.

To address these shortcomings, future studies should incorporate volumetric methods such as confocal z‐stack acquisition, light‐sheet microscopy, or tissue‐clearing strategies coupled with three‐dimensional reconstruction [65]. These approaches would permit a more complete representation of tissue morphology, validate two‐dimensional findings, and provide greater accuracy in mapping epithelial structures and immune interactions.

### 6.3 Limitations and Potential Improvements of Spatial Transcriptomics

Although spatial transcriptomics has transformed transcriptome‐wide analysis within intact sections, several technical challenges persist within mucosal tissue research. A key limitation is resolution: standard platforms often rely on capture areas that encompass multiple cells, requiring computational deconvolution to estimate cell type‐specific expression. Advances on some platforms have however now achieved higher spatial resolution, enabling subcellular transcript localization, along with higher throughput systems to analyze larger tissue areas and multiple samples simultaneously [66].

Performance is also dependent on tissue preparation; fresh‐frozen material yields optimal results, whereas formalin‐fixed paraffin‐embedded samples generally exhibit reduced RNA quality and sensitivity. Additionally, some platforms detect only polyadenylated transcripts and display a 3′ bias, restricting identification of non‐coding RNAs and isoforms. Data sparsity, particularly for low‐abundance transcripts, constitutes an additional analytical challenge. Improvements in barcoding and molecular capture strategies are increasing sensitivity, allowing reliable detection of low‐abundance transcripts.

Despite the limited application of current platforms for large cohort studies, spatial transcriptomics continues to provide valuable insights, especially when used in combination with high‐resolution imaging and single‐cell approaches. Multi‐modal platforms provide a more holistic view of tissue biology by combining spatial transcriptomics with proteomics, epigenomics, and metabolomics. Progress is also being driven by efforts to improve tissue compatibility and incorporate artificial intelligence for image interpretation and data integration. These developments will continue to support substantial advancements in tissue‐level analysis of immune responses.

## 7 Concluding Remarks

In mucosal immunology, the spatial distribution and cellular origin of expressed genes are as important as the molecules themselves; a given mediator can have distinct biological consequences depending on its location. For instance, elevated protease activity in the superficial cervicovaginal epithelium supports normal cell turnover, but similar activity in deeper strata is linked to inflammation and epithelial damage. Moreover, the expression levels of antiproteases, keratins, and epithelial junction proteins follow a structured spatial pattern across the stratified epithelium, which reflects normal keratinocyte differentiation. Disruption or alteration of this protein expression pattern may lead to abnormal mucosal function, which can contribute to disease processes.

Spatial analysis of mucosal genital tissues provides insights that extend well beyond epithelial integrity. By resolving the location, activation state, and interactions of immune cells within their native tissue architecture, spatial approaches could illuminate how HIV‐susceptible target cells accumulate in specific microenvironments, how inflammatory niches form, and how tissue‐resident immune populations contribute to protection or vulnerability. These methods could also reveal how stromal, vascular, and neural components shape local immune dynamics, influencing viral access, dissemination, and early immune responses. Such multi‐layered spatial information advances our understanding of HIV pathogenesis by linking cellular positioning and microenvironmental context to the earliest events of viral transmission and early spread. These processes cannot be captured by bulk or single‐cell analyses alone.

Advances in multi‐omics approaches are enabling increasingly comprehensive views of mucosal tissues by combining spatial, transcriptional, proteomic, and cellular information. At the same time, rapid improvements in computational and analytical pipelines are making it possible to integrate and interpret these complex datasets with greater precision and scalability. Because an intact mucosal immune barrier is essential for protection against sexually transmitted pathogens such as HIV, spatially informed methodological advances offer valuable guidance for developing interventions that preserve epithelial integrity, reduce harmful inflammation, and strengthen localized immune defenses. These approaches can also support the improved design and safety evaluation of topical microbicides and pre‐exposure prophylaxis (PrEP) formulations by revealing how candidate interventions affect mucosal structure, signaling, and cellular composition within relevant tissue compartments.

## Ethics Statement

The authors confirm that the ethical policies of the journal, as noted on the journal's author guidelines page, have been adhered to and the appropriate ethical review committee approval has been received. The study conformed to the US Federal Policy for the Protection of Human Subjects.

## Data Availability

The data that support the findings of this study are available from the corresponding author upon reasonable request.
